# Factors Associated With *Plasmodium falciparum* Infection Transmission in Artisanal Mining and Mining-Free Communities: The Case of Fanteakwa South District, Ghana

**DOI:** 10.1155/japr/6652071

**Published:** 2025-11-03

**Authors:** Enoch Aninagyei, David Adedia, Gifty Larbi, George Abeiku Abbew, Isaac Tukwarlba, Comfort Addo Boatey, Benjamin Sarfo-Bempong, Desmond Omane Acheampong

**Affiliations:** ^1^Department of Biomedical Sciences, School of Basic and Biomedical Sciences, University of Health and Allied Sciences, Ho, Ghana; ^2^Department of Basic Sciences, School of Basic and Biomedical Sciences, University of Health and Allied Sciences, Ho, Ghana; ^3^Department of Biomedical Sciences, School of Allied Health Sciences, University of Cape Coast, Cape Coast, Ghana; ^4^Laboratory Unit, Bososu Health Centre, Fanteakwa South District, Ghana Health Service, Bososu, Ghana

**Keywords:** artisanal mining, asymptomatic malaria, Fanteakwa South District, Ghana, *Plasmodium falciparum*

## Abstract

Artisanal mining in the Fanteakwa South District (FSD) of Ghana has created stagnant water bodies that promote mosquito breeding. This study examined *Plasmodium falciparum* transmission dynamics in mining and nonmining communities. Using stratified sampling, three mining communities and one nonmining community were selected. Blood samples (2 mL) were collected from residents, dried blood spots were prepared, and *P. falciparum* ssrRNA was detected through nested PCR. The study enrolled 341 participants, including 240 (70.4%) from artisanal mining communities and 101 (29.6%) from a mining-free community. Overall, asymptomatic *P. falciparum* prevalence was 53.1%, significantly higher in mining communities (64.6%) than in the mining-free community (27.7%, *p* < 0.001). In mining areas, risk factors included being female (AOR = 2.23, *p* = 0.004), unmarried adult (AOR = 3.87, *p* < 0.001), schooling (AOR = 2.32, *p* = 0.005), not having mosquito nets (AOR = 2.3, *p* = 0.0024), using nets from open markets (AOR = 4.2, *p* = 0.001), net sharing (*AOR* = 3.0, *p* = 0.019), especially three people per net (AOR = 4.9, *p* = 0.024), and staying outdoors at night for >2 h (AOR = 8.4, *p* < 0.001). In the mining-free community, transmission was associated with being a child (AOR = 7.0, *p* = 0.024), schooling (AOR = 3.48, *p* = 0.014), using open-market nets (AOR = 5.67, *p* = 0.037), net sharing (AOR = 4.7, *p* = 0.028), and poor housing quality (AOR = 4.7, *p* = 0.003). The high odds ratios observed for factors such as prolonged outdoor night-time exposure, net sharing, use of substandard nets, and poor housing highlight concentrated risks for *P. falciparum* transmission. In addition, they signal priority areas where targeted interventions, such as quality-assured ITN distribution, behaviour change communication, and housing improvements, would have the greatest impact.

## 1. Introduction

Artisanal mining is a type of small-scale mining (SSM) where no or low mining technology is involved but is characterized by labour-intensive mineral extraction and processing [1]. In all sites with artisanal mining, operators use simple tools such as pickaxes, shovels, pans, and adhesive cotton materials to harvest these natural resources. Artisanal miners are mostly not indigenous, with little or no training in mining activities [2]. Most artisanal miners mine gold, diamonds, tin, and cobalt [3]. However, in Ghana, gold is mostly mined [4]. In Ghanaian parlance, artisanal mining, which is also known as SSM, is referred to as galamsey (which was coined from *gather and sell*) [5]. Artisanal mining in Ghana is largely unregulated and common in several communities and regions [6]. As of April 2022, artisanal mining activities were reported in 14 of the 16 regions of Ghana, with only the Volta and Greater Accra regions unaffected. The Ashanti, Eastern, and Western regions are heavily affected [7].

The high financial incentives provided by miners encourage many landowners to dispose of their parcels of land in pursuit of immediate monetary gain. This practice, however, contributes significantly to the depletion of arable land in Ghana, with implications for agricultural productivity and long-term food security [8]. Surface mining causes serious environmental problems, as evidence of deforestation, land degradation, and water pollution is commonly seen in sites with artisanal mining. In some parts of Ghana, these mining activities are carried out very close to human settlements [9–11]. The degraded lands leave several pockets of stagnant water in these sites. The situation is worse in the rainy season—an occurrence that sustains the life cycle of mosquitoes. Stagnant water promotes the breeding of mosquitoes in mining areas [12–14]. To make matters worse, some studies have reported that mosquitoes found in surface mining sites are resistant to commonly used insecticides [15, 16].

Artisanal mining activities are widespread in the Fanteakwa South District (FSD) in the Eastern Region of Ghana [17]. About 75% of the communities are overtaken by artisanal miners [17]. Artisanal mining activities often result in extensive environmental degradation, including the creation of ponds, ditches, and stagnant water bodies. These water collections serve as ideal breeding habitats for *Anopheles* mosquitoes, the primary vectors of *Plasmodium falciparum*. Consequently, the environmental disturbances caused by artisanal mining directly enhance mosquito proliferation and sustain higher levels of malaria transmission in affected communities. A previous study showed that the rapid diagnostic test (RDT) prevalence of asymptomatic malaria in the district was 43.4% [18]. The study found that people residing in communities with artisanal mining were twice as at risk of asymptomatic malaria compared to people living in mining-free communities. Despite the above, the factors that drive malaria transmission in artisanal mining communities have not been evaluated in the FSD. Therefore, this study uncovered the role artisanal mining plays in the transmission of malaria in the FSD in Ghana.

## 2. Methods

### 2.1. Study Design

A comparative cross-sectional study was conducted to assess the prevalence and the factors that drive malaria transmission in areas with artisanal and nonartisanal mining.

### 2.2. Description and Location of Study Sites

**Figure 1:**
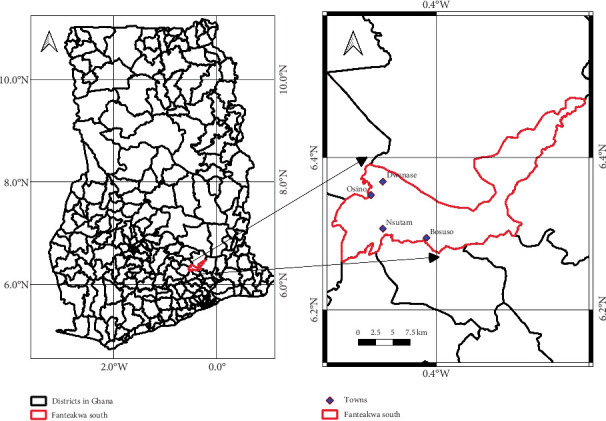
The map of Fanteakwa South District showing the study communities.

This study was carried out in the FSD in the Eastern region of Ghana (Figure 1). The FSD was carved out of the Fanteakwa District in 2018, with Osino as the district capital. The district is located in the central part of the Eastern Region of Ghana. It lies within longitudes 0032.5⁣′ west and latitudes 6015⁣′ north and 6010⁣′ south. The district shares boundaries with Kwahu South District to the north, to the west by Fanteakwa North District, to the south by Abuakwa South District, and to the east by Atiwa East District. The district has a total land area of 460 km^2^. The current population of the FSD is 54,634. Males constitute 50.5% (27,582), while females constitute 49.5% (27,052). The district is predominantly rural. Since the majority of the communities have populations of fewer than 5000, they are classified as rural according to Ghana's population criteria [19]. The population density for the district is 109 people/km^2^ of land (https://fsda.gov.gh/profile/). The district has a longer rainy season than a dry season. Most of the residents are farmers, miners, students, and a few public servants. Artisanal mining is carried out in about three-fourths of the communities in the district. Because of that, the study communities were made up of three communities with artisanal mining activities and a mining-free community. The study communities were randomly selected by using the Epitools application. The application was instructed to randomly select two communities from a list of communities where mining takes place and one community from a mining-free community. The study communities with artisanal mining were Osino (latitude and longitude: 6.3476° N, −0.4868° W), Dwenase (latitude and longitude: 6.3662° N, −0.4679° W), and Nsutam (latitude and longitude: 6.3065° N, −0.4695° W), while the Bososu community (latitude and longitude: 6.2941° N, −0.4129° W) is without artisanal mining.

### 2.3. Number of Participants Studied

The minimum number of participants studied was 303. The number was calculated using Cochrane's formula, *N* = *z*^2^ *p*(1 − *p*)/*e*^2^, where *N* is the sample size, *z* is the standard value of 1.96 corresponding to the confidence level at 95%, and *e* is the error margin at 5%. The prevalence (*p*) of asymptomatic malaria in a contiguous district, Fanteakwa North, used to calculate the sample size was 27% [20], due to the unavailability of such data in the FSD. At each site, a minimum of 76 community members were selected.

### 2.4. Study Population, Selection, and Duration

The population studied in this publication was community members who had stayed at the study sites for more than a year. From each community, about 100 households were randomly selected, from which one person was selected for testing. Household residents were required to pick cards marked ‘Yes' and ‘No'. The person who selected ‘Yes' was recruited for the study. The accompanying adults picked the cards for children younger than 5 years of age. Study participants were recruited from July to October 2023.

### 2.5. Inclusion and Exclusion Criteria

Participants included in the study were those who gave their written consent to participate. In addition, the person must have stayed in the community for more than a year. The study excluded participants with current or past clinical malaria, as well as those who declined to participate.

### 2.6. Study Variables

The dependent variable was the presence or absence of malaria in the community members. However, the independent variables were the presence or absence of mining activities, demographic characteristics, and malaria control practices, including assessment of housing quality. Study variables were obtained through a structured questionnaire. Housing quality was assessed by checking for the presence of ceilings, windows fitted with mosquito nets, the presence of open roof eaves, and regular use of electric fans. These specific housing indicators were selected because they directly influence human–vector contact within households. The presence of ceilings and screened windows reduces mosquito entry, while open roof eaves are known entry points for *Anopheles* mosquitoes. Regular use of electric fans can further deter mosquito biting by creating airflow that disrupts host-seeking behaviour.

### 2.7. Blood Sample Collection

Two millilitres of whole blood was collected from each consented participant. Before blood sample collection, visible antecubital fossa veins were disinfected with 70% alcohol followed by a povidone–iodine solution. In participants without palpable veins and children under 5 years of age, capillary blood was collected from the fingertip, into a tube containing CPDA-1 anticoagulant in a ratio of one part of the anticoagulant to seven parts of blood [21]. Blood samples (50 *μ*L) were used to prepare dried blood spots (DBSs). The DBS samples were stored at room temperature before further analysis.

### 2.8. Molecular Detection of *P. falciparum* in the DBSs

#### 2.8.1. Pre-Extraction Processing of DBSs

A sample haemolysate was prepared by punching two 6 mm DBS into 2.0-mL microcentrifuge tubes. Exactly 1 mL of freshly prepared 0.5% saponin in 1× PBS was added to each tube to completely soak the filter paper. The setup was vortexed and incubated at 4°C overnight to obtain the haemolysate.

#### 2.8.2. *P. falciparum* Nucleic Acid Extraction Using the Chelex Method

After the overnight incubation, the haemolysate, together with the filter paper, was centrifuged at 12,000 rpm for 2 min, and the supernatant comprising saponin and the debris was evacuated by suction. Later, 1 mL of 1× PBS was added to each sample and was centrifuged at 12,000 rpm for 2 min. The process was repeated until there was no haem (red colour) seen in the sample tubes. Subsequently, 50 *μ*L of 20% Chelex suspension and 100 *μ*L of nuclease-free water were added to each sample and heated at 96°C for 10 min to detach any nucleic acids into the Chelex solution. Finally, the setup was centrifuged at 5000 rpm for 5 min, and 120 *μ*L of the supernatant containing any nucleic acids was transferred into a newly labelled microcentrifuge tube, which was stored at −20°C until PCR analysis. The purity and quantity of the nucleic acids were checked with the Nanodrop (Thermo Fisher Scientific Inc., United States).

#### 2.8.3. Molecular Detection of *Plasmodium falciparum*

*P. falciparum* infection status was confirmed by using nested PCR to detect the small subunit rRNA gene of *P. falciparum*. The primers used for the first PCR were rPLU6 (5⁣′-TTAAAATTGTTGCAGTTAAAAC-3⁣′) and rPLU5 (5⁣′-CCTGTTGTTGCCTTAAACTTC-3⁣′) and, subsequently, *P. falciparum*–specific primers rFAL1 (5⁣′-TTAAACTGGTTTGGGAAAACCAAATATATT-3⁣′) and rFAL2 (5⁣′-ACACAATGAACTCAATCATGACTACCCGTC-3⁣′). The first run was made up of 6.25 *μ*L OneTaq Quick-Load 2X Master Mix (New England Biolabs, Ipswich, Massachusetts), 0.25 *μ*L each of 1 *μ*M forward and reverse primers, 4 *μ*L DNA template, and nuclease-free water (1.75 *μ*L) to make 12.5 *μ*L. The *Plasmodium* ssrRNA gene was amplified using the following conditions: 24 cycles of 95°C, 1 min (denaturation); 55°C, 2 min (annealing); and 72°C, 5 min (extension). The second run PCR was made up of 6.25 *μ*L of OneTaq Quick-Load 2X Master Mix, 0.2 *μ*L each of 22 *μ*M forward and reverse primers, 1 *μ*L first run DNA amplicon template, and nuclease-free water to make a 10 *μ*L volume. The *P. falciparum* gene was amplified using these conditions: 35 cycles of 95°C, 1 min (denaturation); 55°C, 2 min (annealing); and 72°C, 5 min (extension). A final band size of 205 bp was diagnostic of *P. falciparum.*

### 2.9. Statistical Analysis

The results were reported using tables and figures that contained frequencies and percentages. A chi-square test or Fisher's exact test was used to assess the association between malaria status and its possible factors among those living in areas with and without mining activity. A multiple logistic regression model was used to determine factors associated with *P. falciparum* infection transmission in mining and mining-free communities while controlling for other variables. A test with a *p* value of at most 0.05 was considered significant.

### 2.10. Ethical Issues

The study was approved by the Ghana Health Service Ethics Review Committee (GHS-ERC: 007/02/22). Written consent was obtained from each study participant.

## 3. Results

### 3.1. Participant Distributions and Their Sociodemographic Variables

A total of 341 participants were selected from four study communities. Of these, 240 (70.4%) came from artisanal mining areas: 70 from Dwenase, 84 from Nsutam, and 86 from Osino, while 101 (29.6%) were from the mining-free community, Bososu. Adults formed the majority in both settings (49.6% in mining vs. 56.4% in nonmining), and females outnumbered males (60.7% vs. 63.4%). In mining communities, most participants were Akan (68%), compared with 50.5% in Bososu. While participants in mining communities were mainly below marital age (43.7%), married individuals were more common in the mining-free community (40.6%). Further details are shown in Table 1.

### 3.2. The Burden of Asymptomatic Malaria in Artisanal Mining and Mining-Free Communities

**Figure 2:**
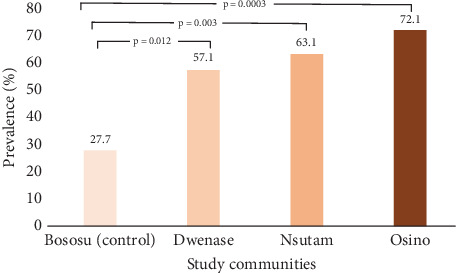
Prevalence of asymptomatic malaria among communities with and without artisanal mining.

Overall, the prevalence of asymptomatic malaria in the study district was 53.1% (183/341). When comparing the prevalence of asymptomatic malaria across the study communities, the rate was significantly lower among participants in Bososu, a community free from mining (27.7%), compared to other mining communities: Dwenase (57.1%, *p* = 0.012), Nsutam (63.1%, *p* = 0.003), and Osino (72.1%, *p* = 0.0003) (Figure 2). It was also determined that in the communities with artisanal mining, asymptomatic malaria was significantly higher in adults (73.1%, *p* = 0.015) compared to children in the mining-free community (83.3%, *p* < 0.001). Furthermore, the prevalence was significantly higher among females in mining sites (60.7%, *p* = 0.003); however, in the mining-free community, asymptomatic malaria was not associated with gender (*p* = 0.421). While asymptomatic malaria was not linked to marital status in mining-free communities, in those with mining, the prevalence was significantly higher among unmarried adults (77.4%, *p* = 0.002). Though the prevalence was higher among schoolchildren in the mining communities (45.8%, *p* = 0.001), in the mining-free community, it was higher among participants who had completed a certain level of education (71.3%, *p* = 0.003) (Table 1).

### 3.3. Demographic Factors Driving Asymptomatic Malaria in Mining and Mining-Free Communities

In mining communities, females were significantly at a higher risk of asymptomatic malaria compared to males (AOR = 2.23, 95% CI: 1.29–3.83, *p* = 0.004). In addition, the odds of having asymptomatic malaria were significantly higher in unmarried adults compared to married people (AOR = 3.87, 95% CI: 1.81–8.26, *p* < 0.001). Further, study participants who were still schooling were also at higher risk of having asymptomatic malaria compared to preschoolers (AOR = 2.32, 95% CI: 1.28–4.22, *p* = 0.005). It was also found that school children in communities with artisanal mining were more than two times at risk of asymptomatic malaria compared to participants who had completed school (AOR = 2.32, 95% CI: 1.28–4.22, *p* = 0.005) (Table 1). In a mining-free community, children under 13 years old were seven times at risk of asymptomatic malaria compared to adolescent participants (13 to 17 years) (AOR = 7.0, 95% CI: 1.29–37.91, *p* = 0.024). However, adults (18 to 65 years) were associated with reduced odds of having asymptomatic malaria (AOR = 0.18, 95% CI: 0.04–0.69, *p* = 0.013). Like the situation in communities with artisanal mining activities, participants who were still in school were at higher odds of contracting asymptomatic malaria compared to participants who had completed school (AOR = 3.48, 95% CI: 1.28–9.43, *p* = 0.014) (Table 1).

### 3.4. Malaria Transmission Factors that Increase the Odds of Having Asymptomatic Malaria in Artisanal Mining and Mining-Free Communities

In communities with artisanal mining activities, 147 (53.7%) participants did not have a mosquito net. Not having a mosquito net was associated with higher odds of having asymptomatic malaria (AOR = 2.3, 95% CI: 1.35–3.99, *p* = 0.0024). Of the 93 (46.3%) participants who had mosquito nets, 42 (45.2%) bought them from the open market. The use of such nets increased the odds of having asymptomatic malaria (AOR = 4.2, 95% CI: 1.75–10.13, *p* = 0.0013) compared to participants who obtained their nets from a healthcare provider. Interestingly, 64 (68.8%) of participants with mosquito nets shared them with others when sleeping. This practice increased the odds of having asymptomatic malaria (AOR = 3.0, 95% CI: 1.20–7.73, *p* = 0.0188) compared to participants who did not share their nets with others. Of the 64 participants who shared their nets, when three people slept in the net, their odds of having asymptomatic malaria were significantly higher (AOR = 4.9, 95% CI: 1.23–19.60, *p* = 0.0243) compared to when two people slept in the nets. In communities with artisanal mining, participants who stayed outdoors at night for over 2 h had higher odds of having asymptomatic malaria (AOR = 8.4, 95% CI: 4.14–17.14, *p* < 0.001) compared to those who stayed outdoors at night for less than 2 h (Table 2). In the community without artisanal mining, even though mosquito net ownership was not associated with asymptomatic malaria status (*p* = 0.161), sharing mosquito nets increased the odds of having asymptomatic malaria (AOR = 8.4, 95% CI: 4.14–17.14, *p* < 0.001) compared to participants who used their nets alone. However, the risk is the same, irrespective of the number of people that slept in the net (*p* = 0.192). Furthermore, as was seen in the site with artisanal mining, purchasing mosquito nets from the open market (AOR = 5.67, 95% CI: 1.11–29.21, *p* = 0.037) increased the risk of having asymptomatic malaria. Exclusive to the community without artisanal mining, having a house of undesirable quality increased the risk of having asymptomatic malaria (AOR = 4.7, 95% CI: 1.7–12.9, *p* = 0.0028) compared to having a site of desirable housing quality (Table 2).

## 4. Discussion

This research work found that the overall prevalence of asymptomatic malaria in the FSD was 53.1%. The observed prevalent rates of asymptomatic malaria in the communities with artisanal mining (Osino 72.1%, Nsutam 63%, and Dwenase 57.1%) were significantly higher than the rate observed in the community without artisanal mining (Bososu 27.7%). This study was conducted over a relatively short sampling period (July–October 2023), which coincided with the peak malaria transmission season in Ghana. While this period provided valuable insights into the prevalence and risk factors of asymptomatic malaria, it may not fully represent the year-round transmission dynamics. Malaria prevalence and mosquito breeding vary seasonally, with transmission intensity generally lower during the dry season. Therefore, the findings might have overestimated the true annual burden of asymptomatic malaria in both artisanal mining and mining-free communities. A longer sampling duration covering both rainy and dry seasons would provide a more comprehensive picture of transmission dynamics across the year.

Assessment of artisanal mining communities revealed major environmental modifications, including abandoned pits, trenches, and stagnant pools of water in Osino, Saamang, Dwenase, Gyampomani, Ankaase, Juaso, Abompe, Nsuapemson, Nsutam, Asamang-Tamfoe, and Bunso. Miners also dammed rivers, leaving stagnant stretches that created mosquito habitats, unlike Bososu, Hemang, and Adjeikrom, which had no artisanal mining. Such alterations have been associated with increased *Plasmodium falciparum* carriage [22–24].

Because mining occurred near settlements, mosquito populations were high around dwellings, though species composition was not confirmed. While *Anopheles* mosquitoes typically breed in clear water [25, 26], recent studies show adaptation to polluted habitats [27–29]. For example, *An. culicifacies* in Sri Lanka and several species in Tanzania and Ghana breed in contaminated water [29–31]. These findings suggest that *Anopheles* increasingly exploit habitats created by mining.

Behavioural and demographic variables strongly influenced asymptomatic malaria risk. Odds were higher among females (AOR = 2.3), unmarried adults (AOR = 4.2), schoolchildren, individuals without nets, those buying nets from open markets, persons sharing nets, and participants engaged in nocturnal activity (>2 h). Notably, nocturnal activity increased odds 8.4-fold, reflecting evening returns from mining and women's extended trading activities [32–34]. Women's higher risk is likely linked to outdoor commerce, while unmarried adults may have irregular sleep [35, 36]. As *Anopheles* in Ghana are predominantly night-biting and photophobic [37–39], extended outdoor exposure increases vulnerability.

Finally, malaria risk was linked to the net source. Although insecticide-treated nets are protective ([40–42]), participants using nets from open markets were 4.2 times more likely to have asymptomatic malaria than those receiving nets from healthcare facilities, reflecting possible differences in net quality, insecticide treatment, and health education.

The higher odds of asymptomatic malaria among participants using nets obtained outside healthcare channels may reflect differences in quality and usage. Nets distributed by health facilities are typically insecticide-treated LLINs and often come with education on correct use, while those from markets or informal vendors may be untreated, counterfeit, or second-hand, reducing their effectiveness. Improper usage and faster wear and tear may also compromise protection. This highlights the need to ensure not only widespread net coverage but also the provision of quality, insecticide-treated nets with adequate user guidance.

The odds of asymptomatic malaria were three times higher when mosquito nets were shared compared to when they were used individually. Sharing among three occupants increased the odds further to 4.9. This suggests that net sharing compromises protective efficacy, likely because nets designed for single users lose their structural integrity when overstretched, creating entry points for mosquitoes. To address this, nets must be obtained from accredited sources and distributed in sufficient quantities to cover every household member. In Ghana, current distribution strategies prioritize children [43] and pregnant women [43]. Expanding coverage to all household members could substantially reduce malaria transmission in endemic communities.

In the nonmining community, transmission appeared to be driven largely by age and socioeconomic factors. Children under 12 years were seven times more likely to harbour asymptomatic malaria compared with adolescents and adults, consistent with the concept of partial immunity in younger age groups that allows them to carry parasites without clinical symptoms [20]. Unemployment also emerged as a risk factor. Unemployed individuals may be unable to afford preventive measures [44], have poorer access to healthcare [45], exhibit suboptimal nutritional status [46], and spend more time outdoors [47], thereby increasing their vulnerability to mosquito bites and malaria transmission.

Poor housing quality further exacerbated transmission risk. Houses without ceilings, unfitted windows, and a lack of fan use at night provide easy access for mosquitoes, a relationship reported in other African settings [48–50].

Although this study focused on asymptomatic malaria, the findings have important public health implications. Individuals with asymptomatic infections serve as reservoirs for transmission [51, 52], and many cases of clinical malaria begin with an asymptomatic stage. Consequently, artisanal mining and its associated environmental and behavioural factors may sustain malaria transmission while also contributing to the overall burden of clinical disease.

The study observed that asymptomatic malaria prevalence was significantly higher among school-aged children in mining communities, while in the mining-free community, it was higher among participants who had completed a certain level of education. This divergence may reflect behavioural differences linked to schooling. In mining communities, schools are often located close to artisanal mining sites where stagnant water bodies and gullies create abundant mosquito breeding grounds. Schoolchildren in these areas spend considerable time outdoors in the early morning and evening hours, when *Anopheles* mosquitoes are most active, thereby increasing exposure to infectious bites. Additionally, the high levels of net sharing reported among school-aged children may further increase their vulnerability. In contrast, in the mining-free community, individuals who had completed schooling, often young adults, may engage in social and economic activities that keep them outdoors at night, such as trading, farming-related work, or leisure gatherings. These behaviours increase exposure to mosquito bites compared to children, who are more likely to be indoors and under supervision at night. Thus, while school attendance appears to increase risk in mining communities due to environmental proximity to mosquito breeding sites, in the nonmining setting, behavioural patterns among older, postschooling individuals seem to drive transmission.

### 4.1. Limitations

The frequencies of some variables were very low, which may have influenced the strength of the associations observed. Furthermore, mosquito populations in the study sites could not be compared, limiting the ability to confirm whether artisanal mining contributed to increased mosquito abundance. The cross-sectional design also precludes establishing causal relationships. Finally, the inclusion of only a single nonmining community as the control group may limit the generalizability of the findings.

### 4.2. Conclusions

This study shows that artisanal mining creates ecological and behavioural conditions that sustain asymptomatic malaria transmission, while nonmining communities are mainly affected by age, socioeconomic status, and housing quality. Key risk factors included female gender, unmarried status, schooling, nocturnal activity, and the use or sharing of substandard mosquito nets. These findings highlight the need to expand equitable distribution of quality insecticide-treated nets to all household members, improve housing, and address socioeconomic vulnerabilities. Given that asymptomatic carriers maintain transmission and contribute to clinical disease, targeted, context-specific interventions are essential to reduce malaria burden and support elimination efforts in endemic regions. To reduce malaria transmission in both mining and nonmining communities, control strategies should go beyond current approaches targeting children and pregnant women. Policies must ensure universal access to quality insecticide-treated nets, improve housing standards, and incorporate socioeconomic support for vulnerable groups. Tailored interventions addressing the unique risks posed by artisanal mining are critical for sustaining progress towards malaria elimination.

## Figures and Tables

**Table 1:** Association of asymptomatic malaria with demographic characteristics of study participants.

	**Artisanal mining communities**					**Artisanal mining-free communities**				
	**Malaria outcome**					**Malaria outcome**				
	**Total (%)**	**Positive, ** **N** ** (%)**	**Negative, ** **N** ** (%)**	**χ** ^2^ ** (** **p** ** value)**	**AOR (95% CI [** **p** ** value])**	**Total (%)**	**Positive, ** **N** ** (%)**	**Negative, ** **N** ** (%)**	**χ** ^2^ ** (** **p** ** value)**	**AOR (95% CI [** **p** ** value])**
Developmental stage^a^				8.33 (0.015)					38.54 (<0.001)^d^	
Child	84 (35)	45 (53.6)	39 (46.4)		0.70 (0.32–1.55) [0.381]	18 (17.8)	15 (83.3)	3 (16.7)		7.0 (1.29–37.91) [0.024]
Adult	119 (49.6)	87 (73.1)	32 (26.9)		1.65 (0.76–3.60) [0.205]	71 (56.4)	8 (12.3)	63 (87.1)		0.18 (0.04–0.69) [0.013]
Adolescent	37 (15.4)	23 (62.2)	14 (37.8)		Ref.	12 (11.9)	5 (41.7)	7 (58.3)		Ref.
Gender				8.57 (0.003)					0.64 (0.421)	
Female	143 (60.7)	103 (72.0)	40 (28.0)		2.23 (1.29–3.83) [0.004]	64 (63.4)	16 (25)	48 (75)		
Male	97 (39.3)	52 (53.6)	45 (46.4)		Ref.	37 (36.6)	12 (32.4)	25 (67.6)		
Tribe				4.76 (0.190)^d^					2.01 (0.231)	
Akan	181 (68)	110 (60.8)	71 (39.2)			51 (50.5)	14 (27.5)	37 (72.5)		
Northern descent	19 (7.6)	14 (73.7)	5 (26.3)			7 (6.9)	2 (28.6)	5 (71.4)		
Krobo/Ga Adamgbe	17 (15)	13 (58.5)	4 (23.5)			43 (42.6)	12 (27.9)	31 (72.1)		
Ewe^b^	23 (9.4)	18 (78.3)	5 (21.7)							
Marital status				12.87 (0.002)					2.47 (0.290)^d^	
Married	49 (29)	23 (46.9)	26 (53.1)		Ref.	41 (40.6)	8 (19.5)	33 (80.53)		
Unmarried adults	84 (27.3)	65 (77.4)	19 (22.6)		3.87 (1.81–8.26) [<0.001]	32 (31.7)	10 (31.3)	22 (68.7)		
Below marital age^c^	107 (43.7)	67 (62.6)	40 (37.4)		1.89 (0.95–3.75) [0.067]	28 (27.7)	10 (35.7)	18 (64.3)		
Formal education				14.5 (0.001)					6.44 (0.038)^d^	
Pre-school^e^	32 (13.1)	14 (43.8)	18 (56.2)		0.56 (0.25–1.25) [0.158]	6 (5.9)	2 (33.3)	4 (66.7)		1.90 (0.32–11.38) [0.482]
In school	110 (45.8)	84 (76.4)	26 (23.6)		2.32 (1.28–4.22) [0.005]	23 (22.8)	11 (47.8)	12 (52.2)		3.48 (1.28–9.43) [0.014]
Completed	98 (40.8)	57 (58.2)	41 (41.8)		Ref.	72 (71.3)	15 (20.8)	57 (62.7)		Ref.
Religion									0.05 (0.870)^d^	
Christian	197 (89.7)	128 (65.0)	69 (35.0)	0.65 (0.723)^d^		83 (82.2)	23 (27.7)	60 (72.3)		
Islam	22 (8.8)	15 (68.2)	7 (31.8)			8 (7.9)	2 (25)	6 (75)		
Traditional	21 (1.5)	12 (57.1)	9 (42.9)			10 (9.9)	3 (30)	7 (70)		
Occupation				0.70 (0.704)					3.89 (0.048)	
Government worker^b^	31 (12.9)	18 (58.1)	13 (41.9)							
Self-employed	88 (36.7)	57 (64.8)	31 (35.2)			69 (68.3)	15 (21.7)	54 (78.3)		Ref.
Unemployed^f^	121 (50.4)	80 (66.1)	41 (33.98)			32 (31.7)	13 (40.6)	19 (59.4)		2.46 (0.99–6.11) [0.052]

Abbreviation: AOR = adjusted odds ratio.

^a^Child (1–12 years), adolescent (13–17 years), adult (18–65 years), and older adult (>65 years).

^b^Variables absent in the study site.

^c^Below marital age, if <18 years.

^d^Fischer's exact test.

^e^Below the formal education age (<4 years).

^f^Includes children under employable age.

**Table 2:** Factors associated with malaria transmission in mining and mining-free communities.

	**Presence of artisanal mining**					**Absence of artisanal mining**				
	**Malaria outcome**					**Malaria outcome**				
	**Total (%)**	**Positive, ** **N** ** (%)**	**Negative, ** **N** ** (%)**	**χ** ^2^ ** (** **p** ** value)**	**AOR (95% CI [** **p** ** value])**	**Total (%)**	**Positive, ** **N** ** (%)**	**Negative, ** **N** ** (%)**	**χ** ^2^ ** ( ** **p** ** value)**	**AOR (95% CI [** **p** ** value])**
Mosquito net ownership				9.39 (0.002)					1.96 (0.161)	
Yes	93 (46.3)	49 (52.7)	44 (47.3)		Ref.	65 (64.4)	15 (23.1)	50 (76.9)		
No	147 (53.7)	106 (72.1)	41 (27.9)		2.3 (1.35–3.99) [0.002]	36 (35.6)	13 (36.1)	23 (63.9)		
Source of mosquito net				10.8 (0.001)					5.13 (0.023)	
Health service provider	51 (54.8)	19 (37.3)	32 (62.7)		Ref.	58 (89.2)	11 (19)	47 (81.0)		Ref.
Open market	42 (45.2)	30 (71.4)	12 (28.6)		4.2 (1.75–10.13) [0.0013]	7 (10.8)	4 (57.1)	3 (42.9)		5.67 (1.11–29.21) [0.037]
Mosquito net usage				5.74 (0.017)					5.37 (0.021)^b^	
I use it alone	29 (31.2)	15 (51.7)	14 (48.3)		Ref.	30 (46.2)	3 (10)	27 (90)		Ref.
Shared	64 (68.8)	49 (76.6)	15 (23.4)		3.0 (1.20–7.73) [0.019]	35 (53.8)	12 (34.3)	23 (65.7)		4.7 (1.2–18.7) [0.028]
Number of persons per one mosquito net				5.68 (0.017)					1.69 (0.192)^b^	
Two	34 (53.1)	22 (64.7)	12 (35.3)		Ref.	17 (62.9)	4 (23.5)	13 (76.5)		
Three	30 (46.9)	27 (90)	3 (10)		4.9 (1.23–19.60) [0.024]	18 (37.1)	8 (44.4)	10 (55.6)		
Previous night ITN usage				2.19 (0.138)^b^					2.28 (1.131)^b^	
No	14 (15.1)	12 (85.7)	2 (14.3)			31 (47.7)	9 (38.7)	19 (61.3)		
Yes	79 (84.9)	52 (65.8)	27 (34.2)			34 (52.3)	6 (9.1)	31 (93.9)		
Prolonged (>2 hours) nocturnal outdoor activity				41.23 (*p* < 0.001)					3.79 (0.051)^b^	
Yes	194 (80.8)	166 (85.6)	28 (14.4)		8.4 (4.14–17.14) [<0.001]	29 (28.7)	12 (41.4)	17 (58.6)		
No	46 (19.2)	19 (41.3)	27 (58.7)		Ref.	72 (71.3)	16 (22.2)	56 (77.8)		
Housing quality				3.75 (0.052)					9.81 (0.0017)^b^	
Desirable	141 (58.8)	84 (59.6)	57 (40.4)			47 (46.5)	6 (12.8)	41 (87.2)		Ref.
Undesirable^a^	99 (41.2)	71 (71.7)	28 (28.3)			54 (53.5)	22 (40.7)	32 (59.3)		4.7 (1.7–12.9) [0.003]

Abbreviation: AOR = adjusted odds ratio.

^a^Undesirable housing quality was defined as a house having one or more of these features: absence of ceilings, windows not fitted with mosquito nets, the presence of open roof eaves, and the absence and irregular use of electric fans.

^b^Association tested by Fisher's exact test.

## Data Availability

All data generated are contained in this publication.
